# Development of a mu3ABC-Based Lateral Flow Immunochromatographic Strip for Rapid DIVA-Compatible Detection of Antibodies Specific to Foot-and-Mouth Disease Virus

**DOI:** 10.3390/vetsci13080797

**Published:** 2026-08-11

**Authors:** Wantanee Tommeurd, Vijittra Naithong, Jullada Chootip, Kingkarn Boonsuya Seeyo, Porntippa Lekcharoensuk

**Affiliations:** 1 Department of Microbiology and Immunology, Faculty of Veterinary Medicine, Kasetsart University, Chatuchak, Bangkok 10900, Thailand; w.tommeurd@gmail.com (W.T.); aomsinisasomin@gmail.com (V.N.); jullada.cho.vet77@gmail.com (J.C.); 2 Graduate Program in Animal Health and Biomedical Sciences, Faculty of Veterinary Medicine, Kasetsart University, Chatuchak, Bangkok 10900, Thailand; 3 Regional Reference Laboratory for Foot and Mouth Disease in Southeast Asia, Pak Chong, Nakhon Ratchasima 30130, Thailand; kingkarn.bs3244@hotmail.co.th; 4 Center for Advanced Studies for Agriculture and Food, Kasetsart University Institute for Advanced Studies, Chatuchak, Bangkok 10900, Thailand

**Keywords:** foot-and-mouth disease (FMD), non-structural protein (NSP), lateral flow immunochromatographic assay (LFA), strip test, differentiation of infected from vaccinated animals (DIVA)

## Abstract

Foot-and-mouth disease (FMD) is one of the most infectious viral diseases of cloven-hoofed animals. The crucial strategy for FMD control is the combination of animal quarantine and a rapid onsite diagnostic assay capable of differentiation of infected from vaccinated animals (DIVA). Herein, we have developed a lateral flow immunoassay to detect specific antibodies against FMDV non-structural protein (NSP), based on recombinant mu3ABC produced by *E. coli* as an antibody detector, the so-called mu3ABC strip test. The performance of this test was examined using 366 field serum samples from cattle, pigs, and goats and compared with the commercial ELISA kit, resulting in a diagnostic sensitivity (DSn) and specificity (DSp) of 98.05% and 93.16%, respectively. Additionally, efficacies of this test and commercial ELISA were determined using 200 reference bovine serum samples (100 positives, 100 negatives) classified by the Regional Reference Laboratory for Foot-and-Mouth Disease in South East Asia (RRL). The result reveals that the DSn and DSp of the test were 78% and 91%, respectively, while those for the commercial ELISA kit were 69% and 100%, respectively. The developed mu3ABC strip tests are effective and can be used to differentiate the infected animals in parallel with the sero-surveillance ELISA test.

## Simple Summary

Foot-and-mouth disease is a highly contagious viral illness that threatens livestock such as cattle, pigs, and goats. To control outbreaks effectively, authorities need a fast, onsite tool to distinguish between infected animals and those that have been vaccinated. To address this, we developed a rapid, easy-to-use paper test strip that detects specific proteins produced during an active infection. We evaluated this new test using 366 field animal serum samples and 200 reference samples, comparing its performance against a standard laboratory method. The results showed that our test strip is highly accurate, achieving a diagnostic sensitivity of 98.05% and a diagnostic specificity of 93.16% in field samples, while also performing reliably against reference samples. In conclusion, this newly developed test strip is an effective tool that can be used directly on farms alongside routine surveillance. By enabling rapid identification of sick livestock, this test helps farmers and veterinarians control disease outbreaks quickly, ultimately protecting animal welfare, securing food supplies, and supporting the livelihoods of farming communities directly.

## 1. Introduction

Foot-and-mouth disease (FMD) is a rapidly spread and highly contagious vesicular disease of cloven-hoofed animals, including swine, cattle, buffalo, goat, and sheep. It is distributed across Asia, Africa, and Eastern Europe and has a high economic impact in endemic countries. Its incubation period depends on the species of animal, exposure dose, and the route of infection [1]. Cattle and swine are highly susceptible to foot-and-mouth disease virus (FMDV), while the clinical outcome in small ruminants is mild or asymptomatic [2]. Asymptomatic carrier animals can spread the virus, leading to infection in susceptible animals. The virus remains in epithelial cells and lymphoid tissues in oropharyngeal areas of cattle for 28 days [3]. Deep sequence analysis revealed the presence of FMDV subpopulations including antigenic escape mutants in the nasopharyngeal mucosa of persistently infected cattle [4].

FMDV comprises seven distinct serotypes, O, A, C, Asia 1, SAT 1, SAT 2, and SAT 3, each of which includes various subtypes and topotypes, with no cross-protection among serotypes [5,6]. FMDV is distributed globally and is classified based on VP1 sequences into seven endemic pools of FMDV serotypes and topotypes circulating in specific regions [7]. Pools 1, 2, and 3 encompass distinct geographical areas across Asia and Eurasia (Southeast/Central/East Asia, South Asia, and West Eurasia/Middle East, respectively), of which serotypes O, A, and Asia 1 are predominantly circulating in all three regions. Serotype O is the most prevalent in Asia and present in all countries [8]. However, the circulating FMDV topotypes or lineages in each pool may be different, such as O/SEA/Mya98 in Pool 1 and O/ME-SA in Pool 2, with an occasional incursion from Pool 2 to Pools 1 and 3 [9]. Regarding Africa, the virus distribution comprises three endemic pools: Pool 4 (Eastern and North Africa), which is characterized by the circulation of serotypes O, A, SAT 1, SAT 2, and SAT 3 in Eastern Africa, whereas only serotypes A and O are present in North Africa; Pool 5 (West and Central Africa), which includes serotypes O, A, SAT 1, and SAT 2; and Pool 6 (Southern Africa), which is restricted to serotypes SAT 1, SAT 2, and SAT 3. Lastly, Pool 7 is confined to South America, where serotypes O and A circulate exclusively in Venezuela. Recently, cross-pool migrations of FMDV have been observed across multiple regions [7]. For instance, the O/ME-SA/Ind-2001e lineage (Middle East–South Asia topotype), introduced from Pool 2 to Pool 1 between 2016 and 2021, subsequently became the predominant lineage circulating in Southeast Asia [7]. More recently, the co-circulation of SAT 1 topotype I (originating from Pool 4 in East Africa) and a seed-vaccine-related SAT 1 topotype III strain was detected in the Middle East in 2025 [10,11]. Subsequently, in March 2026, SAT 1 topotype III incursion was reported in Xinjiang and Gansu provinces, China, situated 2000 km apart [12]. This transboundary dissemination across endemic pools and the coexistence of multiple serotypes underscore the critical role of livestock movements in widespread disease transmission.

Animal quarantine and the screening of infected livestock for slaughter are core control strategies that have proven effective in maintaining FMD-free status [13]. However, in FMD endemic areas where vaccination is implemented as a control measure, a significant challenge in these programs is the presence of persistently infected animals, which can act as silent reservoirs of the virus [4,14]. To address this, a diagnostic tool that can differentiate infected from vaccinated animals (DIVA) has become essential [15]. The foundation of the DIVA strategy lies in detecting antibodies against non-structural proteins (NSPs) of the virus, which are produced during viral replication and are typically absent in purified antigen-containing vaccines. Therefore, they act as a specific marker to distinguish immunity raised by natural infection from vaccination. Among the various NSPs evaluated for the DIVA strategy, the 3ABC polyprotein stands out due to its high immunogenicity [16]. Antibodies against 3ABC are detectable shortly after infection and tend to persist longer than those targeting other NSPs [17]. Consequently, the 3ABC-based assay is widely considered the most reliable diagnostic tool for the DIVA strategy [16,17,18,19].

Recently, immunochromatographic strips have been widely adopted across various fields due to their specificity, rapid turnaround, and suitability for field-based detection. In this system, colloidal gold nanoparticles are commonly used as a label to provide a visible signal and are conjugated with specific antibodies or antigens to form a detection complex. As the sample flows across the nitrocellulose membrane via capillary action, the target analytes are captured at the test line, resulting in a visible color change. Several immunochromatographic assays have been developed for detecting and differentiating FMDV antibodies in pig serum samples [20,21]. Previously, we developed an FMDV diagnostic ELISA based on 3ABC for antibody detection and DIVA discrimination [22]. In this study, to enhance user convenience and field adaptability, we developed a 3ABC-based lateral flow immunoassay (LFA) using colloidal gold nanoparticles for rapid visibility. The performance of this assay was demonstrated across three different livestock species using both reference and field sera. This assay provides a practical and rapid tool for FMD surveillance, facilitating the efficient movement control of animals both domestically and internationally.

## 2. Materials and Methods

### 2.1. Serum Samples and Reagents

A total of 566 serum samples from cattle, pigs, and goats were used to evaluate the performance of the mu3ABC strip test. All samples were leftovers of sera collected from livestock farms in Thailand for diagnostic purposes. The first panel consisted of 366 field serum samples obtained from cattle (*n* = 120), pigs (*n* = 137), and goats (*n* = 109), and the second panel comprised 200 reference cattle sera classified as 100 positive and 100 negative serum samples by the Regional Reference Laboratory (RRL) in 2023. Mouse IgG, bovine serum albumin (BSA), casein, sucrose, and Tween-20 were purchased from Sigma–Aldrich Chemical Corporation (St. Louis, MO, USA). All solvents and other chemicals were of analytical reagent grade.

### 2.2. Production of 3ABC Polyprotein

mu3ABC is a genetically engineered form of the FMDV 3ABC gene by substituting cysteine at positions 142 and 163 with serine and glycine, respectively [23]. In this study, mu3ABC cloned in pQE80L (Qiagen, Germantown, MD, USA) was expressed in *E*. *coli* cultures, as described previously [24]. Briefly, BL21 DE3 *E*. *coli*-competent cells (New England Biolabs, Ipswich, MA, USA) were transformed with pQEmu3ABC and induced in early-log phase culture using 0.2 mM IPTG for 4 h. Cultures were collected and the *E*. *coli* cell disruption was achieved by chemical and physical methods. In addition, a recombinant Autographa Californica Multiple Nucleopolyhedrovirus (AcMNPV) containing the mu3ABC gene was produced and inoculated into High Five insect cells (Thermo Fisher Scientific, Waltham, MA, USA) to express mu3ABC as described elsewhere [22]. Both insoluble mu3ABC proteins obtained from *E*. *coli* and insect cells were dissolved in binding buffer (50 mM NaH_2_PO_4_, 8 M Urea, pH 7.5) and loaded into a HiTrap SPFF column (Cytiva, Marlborough, MA, USA), following the manufacturer’s protocol. The eluate fractions with the mu3ABC band in 10% SDS-PAGE were pooled and loaded into the HisTrap FF column (Cytiva, Marlborough, MA, USA). The presence of mu3ABC in the eluates was confirmed by 10% SDS-PAGE and Western blot. The concentration of the purified mu3ABC was measured using the Pierce™ 660 nm protein assay (Thermo Fisher Scientific, Waltham, MA, USA), and the protein was stored at −80 °C until used.

### 2.3. SDS-PAGE and Western Blot

mu3ABC profiles were studied using SDS-PAGE and Western blot following the previously published protocol [24]. The purified mu3ABC was electrophoresed through SDS-PAGE (Thermo Scientific, Waltham, MA, USA) with 10% resolving gel and 4% stacking gel at 140 V and 300 mA for 65 min. The gel was subsequently blotted onto a nitrocellulose membrane (Bio-Rad Laboratories, Hercules, CA, USA) in Tris-Glycine transfer buffer (25 mM Tris, 192 mM glycine, and 20% methanol, pH 9.9) using the wet transfer method. After blocking with BlockPRO™ 1 Min Protein-Free Blocking Buffer (Energenesis Biomedical, Taipei, Taiwan), the blotted membrane was incubated with anti-FMDV serum (1:200) at room temperature for 1 h, followed by incubating with protein G conjugated with HRP (Sigma-Aldrich, St. Louis, MO, USA) diluted at 1:2000 in TBST (50 mM Tris-HCl pH 7.5, 100 mM NaCl, 1 mM EDTA, 0.1% Tween 20). After each step, the membrane was washed with TBST three times. Finally, the membrane was incubated in TMB peroxidase substrate (Sera Care, Milford, MA, USA) until the color was developed. The reaction was stopped by thoroughly rinsing the membrane with deionized water.

### 2.4. Optimization of Essential Parameters

A simple dot blot was performed to optimize conditions for strip test development. The assay performance was evaluated using serum samples diluted at 1:10 and 1:5 in sample buffer. For the capture zones, recombinant mu3ABC protein at concentrations of 0.5, 1, and 2 mg/mL was immobilized on the test (T) zone, while mouse IgG (1 mg/mL) served as the control (C) zone. Furthermore, the protein G-conjugated gold nanoparticles (AuNPs) were evaluated at optical densities (OD520) of 2.8 and 4.2 (2× and 3× concentrations, respectively) to determine the optimal signaling intensity.

To assess signal intensities of the dot blots, three independent replicates (*n* = 3) were quantified using ImageJ software (ImageJ v. 1.54t, National Institutes of Health, Bethesda, MD, USA). Statistical analyses were performed using Microsoft Excel 2016 via the Data Analysis ToolPak, with data expressed as mean ± standard deviation (SD). For two-condition comparisons (amount of protein G-conjugated gold nanoparticles and serum sample dilutions), a two-sample *t*-test assuming unequal variances (one-tailed) was employed based on directional hypotheses. For multi-group comparisons regarding the concentration of mu3ABC, a one-way analysis of variance (ANOVA: Single Factor) was conducted to evaluate variance concurrently and mitigate cumulative Type I errors. In all instances, a *p*-value of less than 0.05 (*p* < 0.05) was considered statistically significant.

### 2.5. Preparation and Assembly of the Strip

The conjugate pad (K Biosciences, Pathumthani, Thailand) was prepared by coating it with protein G-conjugated colloidal gold nanoparticles using an XYZ3000 Dispense system (BioDot, Irvine, CA, USA) at a jetting rate of 10 µL/cm, followed by drying at 37 °C for 2 h.

For the nitrocellulose (NC) membrane (CN140; Sartorius, Gottingen, Germany), mouse IgG (1 mg/mL) and recombinant mu3ABC protein (1 mg/mL) were dispensed at 1 µL/cm to form the control (C) and test (T) lines, respectively, using the same XYZ3000 system. The membranes were then immersed in a blocking solution (1% BSA, 0.05% Tween 20, and 3% sucrose in 0.15 M PBS) for 30 min, and subsequently dried at 37 °C for 1 h.

Finally, the processed NC membrane, conjugate pad, sample pad (Ahlstrom, Helsinki, Finland), and absorbent pad (Cytiva, Marlborough, MA, USA) were assembled onto adhesive backing cards (Kenosha, Amstelveen, the Netherlands) with a 2 mm overlap between adjacent components. The fully assembled cards were then precision-cut into 4 mm wide strips using a CM4000 Guillotine cutter (BioDot, Irvine, CA, USA).

### 2.6. Assay Procedures

Ten-fold serum samples diluted with sample buffer were applied to the sample pad, and after the sample had migrated to the absorbent pad, an additional 70 µL of sample buffer was added to the sample pad as a chaser. The results were visually observed within 20–30 min. The visual interpretation of the lateral flow strips was performed independently by two operators.

### 2.7. Comparison Study of mu3ABC Strip with an FMDV ELISA Kit

To evaluate diagnostic performance, 366 serum samples from cattle, pigs, and goats were tested. Each sample was analyzed using both the mu3ABC strip test and the ID Screen^®^ FMD NSP ELISA kit (Innovative Diagnostics, Grabels, France), the latter being performed according to the manufacturer’s instructions. For ELISA, a sample-to-negative control percentage (S/N%) threshold of ≤50% was used to define positive results. This value represents the ratio of the optical density (OD) of the test sample to that of the negative control, multiplied by 100 to yield a percentage.

### 2.8. Statistical Analysis

Diagnostic sensitivity and specificity were calculated using 2 × 2 contingency tables. The agreement between the mu3ABC strip test and the commercial ELISA was evaluated using Cohen’s kappa coefficient [25], available in the GraphPad QuickCalcs online calculator (GraphPad Software, San Diego, CA, USA). Kappa values were interpreted as 0.00–0.20 (slight), 0.21–0.40 (fair), 0.41–0.60 (moderate), 0.61–0.80 (substantial), and 0.81–1.00 (almost perfect).

## 3. Results

### 3.1. Comparison of mu3ABC Produced in E. coli and Insect Cells

**Figure 1:**
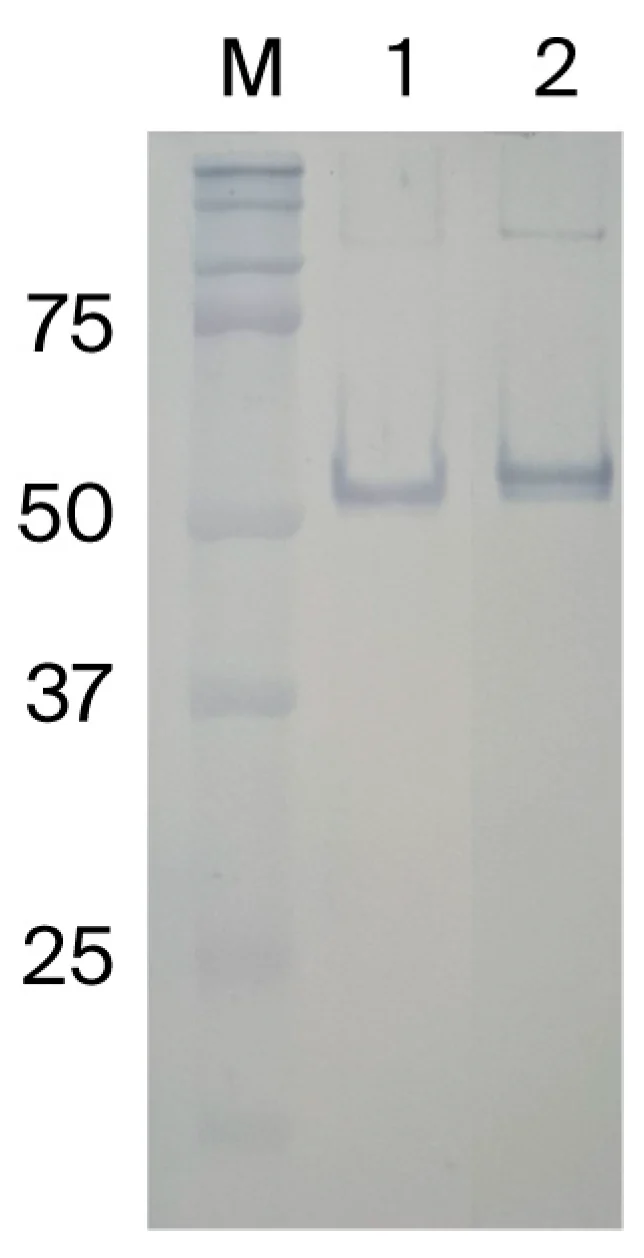
Western blot analysis of the purified mu3ABC with FMDV-positive bovine serum. Lanes 1 and 2 contain purified mu3ABC from *E. coli* and insect cells, respectively.

Previously, we and other studies demonstrated that mu3ABC protein expressed in insect cells was a suitable antigen for development of ELISA to differentiate infected from vaccinated animals [17,19,22]. In this study, we attempted to decrease costs while increasing robust protein production by changing the mu3ABC expression host from insect cells to *E*. *coli*. We found that the recombinant mu3ABC expressed in both *E*. *coli* and insect cells had a molecular weight of approximately 55 kDa. Analysis using SDS-PAGE and Western blot revealed that both *E*. *coli-* and insect-cell-derived mu3ABC were expressed mainly as inclusion bodies within the insoluble fraction. Thus, the solubilization required urea at a concentration of 8 M. Western blot was used to verify the specificity of the purified *E*. *coli-* and insect-cell-derived mu3ABC against anti-FMDV antibodies in a convalescent bovine serum (Figure 1, Figure S1 and Table S1). The results confirmed that mu3ABC produced in insect cells could be replaced with that from *E*. *coli*.

### 3.2. Optimization of Different Parameters

**Figure 2:**
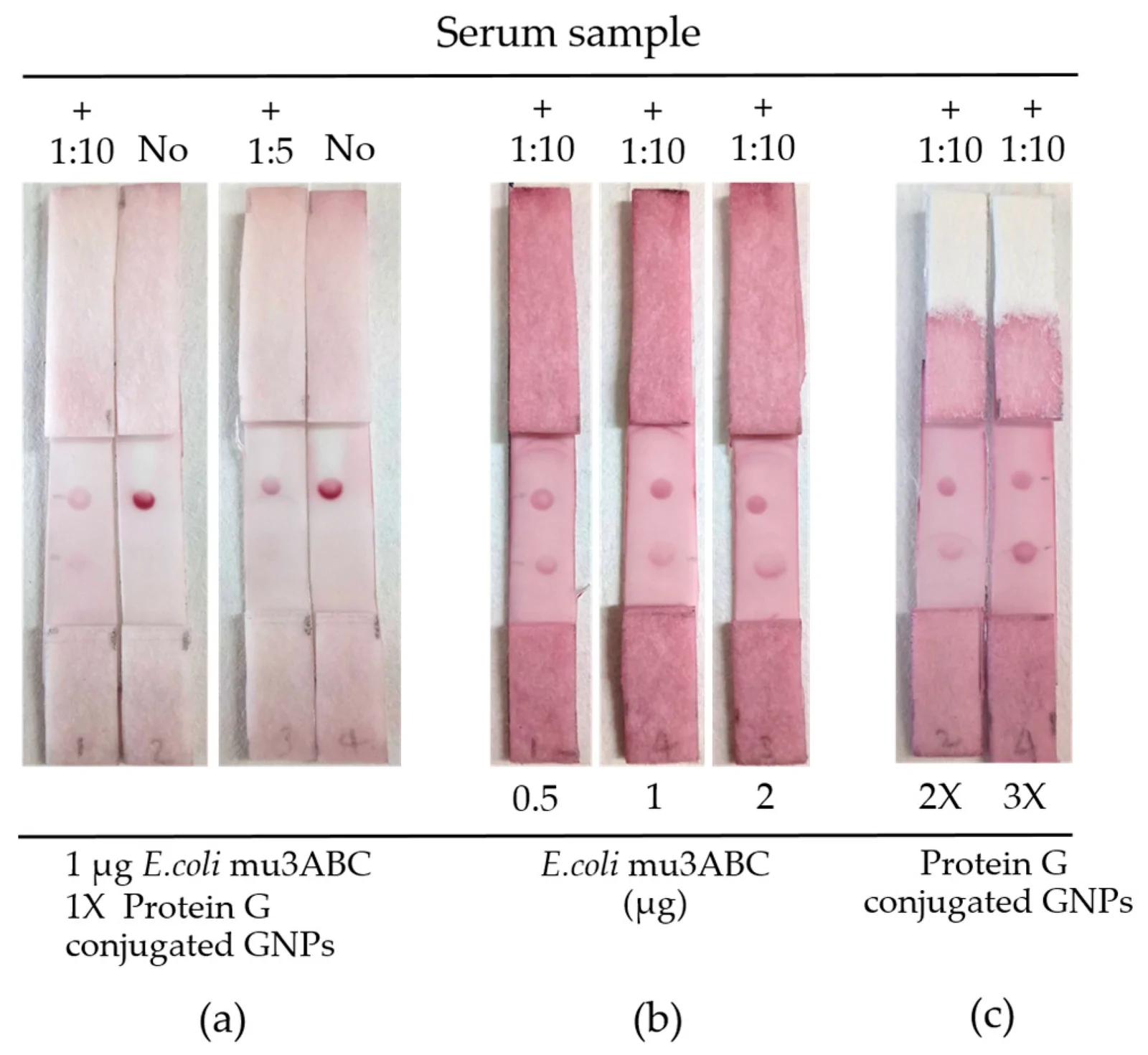
Optimization of key parameters for mu3ABC strip test: (**a**) the dilution of serum sample, (**b**) the concentration of mu3ABC, and (**c**) the amount of protein G-conjugated gold nanoparticles. Upper dot: mouse IgG; lower dot: mu3ABC protein. The image shown is representative of three independent experiments.

Key parameters were systematically optimized to maximize diagnostic sensitivity and minimize background interference. Preliminary evaluations were conducted using hand-assembled test strips to determine the appropriate conditions for subsequent strip production. The optimized factors including the serum sample volume, the concentration of mu3ABC, and the amount of protein G-conjugated gold nanoparticles with all corresponding data and statistical outcomes were compiled in Table S2. First, the evaluation of different serum sample dilutions revealed that the 1:10 dilution provided a significantly clearer signal compared to the 1:5 dilution (*p* = 0.033, one-tailed *t*-test) (Figure 2 and Figure S2a). Consequently, the 1:10 dilution was determined as the optimal working ratio. For the test line, mu3ABC was evaluated at concentrations of 0.5, 1, and 2 mg/mL. A one-way ANOVA indicated that varying the mu3ABC concentration within this range caused no statistically significant changes in signal performance (*p* = 0.917). Based on these findings, 1 mg/mL was chosen as a practical optimum (Figure 2 and Figure S2b). Finally, the varied amount of protein G-gold conjugate was investigated by comparing OD520 of 2.8 (2×) and 4.2 (3×). The 3x concentration yielded a higher mean signal intensity on the test line compared to the 2x concentration (Figure 2, Figure S2c and Table S2). The analysis revealed that the 3x condition yielded a significantly higher mean signal intensity on the test line compared to the 2x condition (*p* = 0.038, one-tailed *t*-test). Furthermore, no statistically significant difference in signal intensity was observed between the control lines of both conditions (*p* = 0.317, one-tailed *t*-test), indicating baseline stability. Based on the significant enhancement of the test line signal, the 3x condition was selected as the optimal amount for the assay.

### 3.3. Diagnostic Performance Across Species

The diagnostic performance of the mu3ABC strip test was evaluated across three animal species, using the commercial NSP ELISA as a reference (Table 1). In swine samples, the strip test demonstrated 100% sensitivity and specificity. A similarly high sensitivity of 100% was observed in goats, although the specificity was slightly lower at 91.51% due to nine ELISA-negative samples yielding positive results on the strip. In cattle, the strip test achieved 96.77% sensitivity and 84.48% specificity, compared to the NSP ELISA.

**Figure 3:**
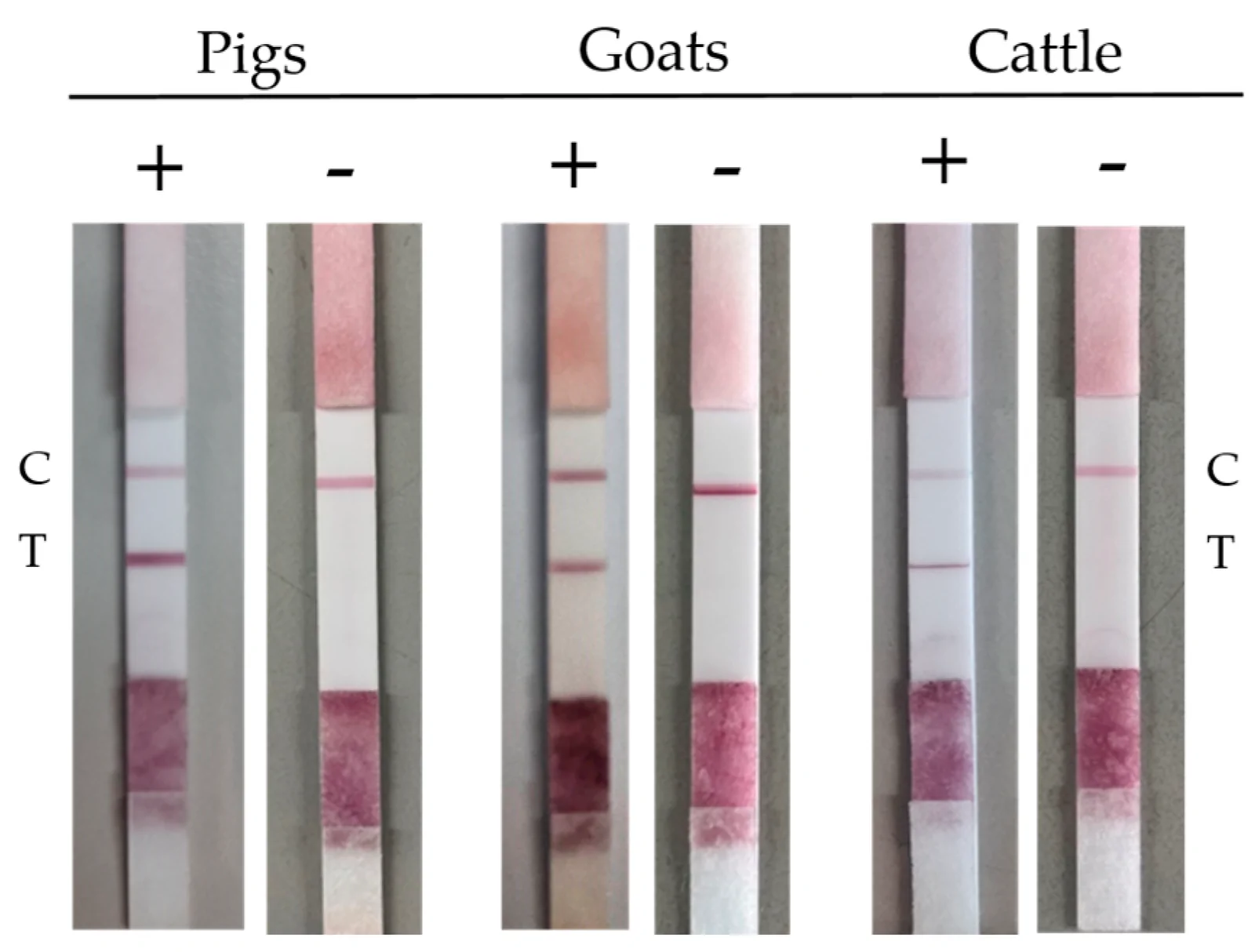
Representative results of the mu3ABC strip test across different livestock species. Symbols (+) and (−) indicate the corresponding results obtained from the commercial NSP ELISA used for comparison. The upper band represents the control line (C), confirming the validity of the assay, while the lower band indicates the test line (T) positive for FMD NSP antibodies.

A total of 366 serum samples from various cloven-hoofed species, including cattle, pigs, and goats, were tested using the mu3ABC strip tests. FMDV-positive sera containing NSP-specific antibodies result in visible red bands at both the test and control lines, while negative sera show a visible band only at the control line (Figure 3). In parallel with the strip test, the sera were tested with the commercial ELISA kit. The sensitivity and specificity of the strip test, relative to the ELISA, were 98.05% (101/103) and 93.16% (245/263), respectively, and the test also showed substantial agreement with commercial ELISA, yielding a Cohen’s kappa value of 0.871 with a 95% CI of 0.816 to 0.926.

### 3.4. Sensitivity and Specificity of Immunochromatographic Strip

To further evaluate the diagnostic performance, the strip test was additionally tested using a panel of 200 reference serum samples from the RRL, classified as positive and negative samples in 2023. The results revealed that the mu3ABVC strip test had a sensitivity of 78% and a specificity of 91% (Table 2). It is important to note that variations in performance among serological platforms can occur due to differences in target epitopes and cutoff thresholds. This was evident as the commercial ELISA used in this study also classified only 69 out of the 100 positive reference samples as positive according to the manufacturer’s instructions. The identical Cohen’s kappa coefficient of 0.690 achieved by both the strip test and the commercial ELISA represents a substantial level of agreement with the reference standard, demonstrating that the mu3ABC strip test provides diagnostic reliability comparable to the established commercial ELISA kits.

## 4. Discussion

Lateral flow immunochromatographic assays are widely used as rapid, cost-effective diagnostic tools for point-of-care testing. The LFA platform consists of three functional elements: an immobilized protein at the control line for assay validation, a specific antigen at the test line for target analyte capture, and a colloidal gold-labeled detector that facilitates visual observation. In a previous study, our team developed a recombinant 3ABC protein in which two cysteine residues at the 3C region (positions 142 and 163) were substituted with serine and glycine, respectively [24]. It was initially expressed using an insect cell system and successfully utilized as a diagnostic antigen in a 3ABC-based ELISA to differentiate infected from vaccinated animals [22]. Consequently, this modified 3ABC protein was selected as the immobilized antigen at the test line in the current study. While the initial production in insect cells took approximately 96 h for expression, the *E*. *coli* expression system required an induction period of only 4 h. Evaluation by SDS-PAGE and Western blot confirmed that the quality and antigenicity of the recombinant proteins were comparable. Furthermore, bacterial culture media are much cheaper and simpler than the media used for insect cell cultivation. Accordingly, the *E*. *coli* system was adopted for mu3ABC production due to its significantly lower cultivation time and cost-effectiveness.

Maximizing the diagnostic performance in this study was achieved through the optimization of physicochemical parameters. Our results demonstrate that adjusting the antigen loading density on the test line, the concentration of the colloidal gold-mu3ABC conjugate, and the optimal sample volume was critical for maximizing binding efficiency. Furthermore, modifying the ionic strength and pH of the running buffer, along with adding surfactants such as Tween-20, significantly reduced non-specific binding and improved the signal-to-noise ratio. These practical optimizations provided a cost-effective route to increasing sensitivity, especially in challenging samples. In addition to these physical parameters, the assay could be further improved at the molecular level, such as employing computational epitope mapping to identify and exclude cross-reactive epitopes within the 3ABC polyprotein [26]. The optimization of protein orientation via site-specific biotinylation could also improve antigen–antibody binding efficiency [27]. With these fundamental parameters established, future integration of digital image-based quantification could serve as a robust form of signal amplification [28,29]. This would further enhance the detection of low-titer samples, providing a sensitive and objective DIVA diagnostic tool for large-scale FMD surveillance across diverse livestock species.

The versatility of the developed LFA is largely attributed to the use of protein G-conjugated gold nanoparticles as a universal detector. Unlike conventional assays that rely on species-specific secondary antibodies, protein G exhibits a high and broad-spectrum affinity for the Fc region of IgG across various species [30,31]. By specifically targeting this portion, the antigen-binding sites remain accessible for efficient binding to the 3ABC antigen. This structural advantage ensures that the formation of the immunocomplex is not compromised, leading to high diagnostic sensitivity and providing the basis for a multi-species diagnostic platform. Since protein G exhibits species-dependent binding affinities for immunoglobulins, systematic optimization of key assay parameters, specifically serum dilution ratio, the immobilized mu3ABC antigen, and the amount of protein G nanoparticles, was essential during method development. Balancing these three variables effectively mitigated binding discrepancies and matrix interferences, allowing the mu3ABC strip test to achieve high diagnostic sensitivity and acceptable to high specificity across pigs, goats, and cattle. In this regard, while previous studies also developed NSP-based LFAs [20,21], their applications were primarily focused on FMDV antibody detection in pig sera. However, our mu3ABC-based LFA provides a broader application across three different livestock species, including cattle, swine, and goats. As a result, this species-independent platform offers a more practical and convenient way to monitor FMDV across different types of livestock.

The observed variations in sensitivity between the mu3ABC strip test and the NSP ELISA could be linked to the different physical environments of the antigen. In the strip test, the antigen is adsorbed and dehydrated on a nitrocellulose membrane matrix, whereas in the ELISA, it remains bound to a polystyrene surface within a buffered aqueous environment. These surface-dependent conformational changes, along with the shorter reaction times inherent to lateral flow assays, may affect epitope accessibility and binding affinity [32,33].

The discrepancy in assay performance when using different diagnostic tests as gold standards is inevitable as no assay possesses 100% accuracy [34,35]. In the first panel, the ID Screen ELISA was used as the gold standard for mu3ABC strip test validation, yielding a diagnostic sensitivity (Dsn) of 98.05% (101/103) and a diagnostic specificity (Dsp) of 93.16% (245/263). When both ID Screen ELISA and mu3ABC ELISA were compared using the 200 reference serum samples previously classified as positive and negative using the third NSP ELISA by RRL, the Dsn and Dsp of mu3ABC ELISA decreased to 78% and 91%, respectively, while those of ID Screen ELISA were 69% and 100%, respectively. Both ID Screen and the third NSP ELISA are competitive (blocking) ELISAs whose classification relies on a single FMDV monoclonal antibody. The cutoff threshold for the ID Screen ELISA appears to have been optimized for high diagnostic specificity resulting in a Dsp approaching 100% alongside a reduced Dsn of 69% on the RRL sample panel. Since animal sera contain polyclonal antibodies targeting diverse epitopes of FMDV non-structural proteins (NSPs), non-competing antibody clones are not blocked by the targeted monoclonal antibody. This single-epitope specificity inherently reduces the assay sensitivity. In contrast, the mu3ABC strip test captures a broader repertoire of anti-NSP polyclonal antibodies, demonstrating higher sensitivity than the ID Screen ELISA. On the other hand, the mu3ABC strip test is a lateral flow immunochromatographic assay. During execution, serum antibodies bind to protein G conjugated with nanogold and migrate rapidly across the nitrocellulose membrane toward the absorbent pad. This rapid migration allows only high-affinity antibody clones to be captured by the coated antigen on the membrane [36], typically conferring lower analytical sensitivity relative to liquid-phase ELISAs. Consequently, the Dsn of the mu3ABC strip test was 78% relative to the highly sensitive RRL reference ELISA. However, the mu3ABC strip test maintained comparable diagnostic specificity across both ELISA reference panels (93.16% vs. 91.00%), reflecting a consistent specificity profile. More importantly, sample aging between 2023 (initial RRL testing) and 2025 (this study) might have also contributed to the observed performance variations. Over this two-year storage period, potential serum degradation prevents direct performance comparison with the baseline. This degradation affected diagnostic sensitivity of both ID Screen ELISA and the mu3ABC strip test. Therefore, these discrepancies reflect the differential impact of sample aging on each platform rather than intrinsic limitations of the mu3ABC assay.

While NSP-based ELISA remains the gold standard for DIVA due to its high sensitivity, it requires specialized laboratory facilities, trained personnel, and time-consuming procedures. In contrast, lateral flow assays (LFAs) provide a rapid, point-of-care solution for field diagnostics. These assays enable immediate screening in resource-limited areas where laboratory access is constrained. In practice, the LFA can be implemented as an initial diagnostic tool for onsite decision making at animal checkpoints or during suspected outbreaks. This facilitates real-time quarantine measures while samples are being transported for laboratory confirmation via ELISA. By combining the speed of the LFA with the high sensitivity of the ELISA, more effective outbreak management can be achieved.

The strategic advantage of the developed mu3ABC strip test lies in its ability to overcome the limitations of molecular diagnostics in long-term surveillance. While RT-qPCR is inherently capable of identifying infected animals, its application in DIVA is limited by the transient nature of viral shedding [37]. In contrast, our LFA identifies long-lasting NSP-specific antibodies, providing a broader diagnostic window even after molecular markers are no longer detectable. Furthermore, when compared to emerging point-of-care (POC) platforms such as RT-LAMP [38,39], CRISPR-based assays [40,41], or electrochemical biosensors [28,42], the mu3ABC LFA remains the most viable option for large-scale screening. Although isothermal and CRISPR methods offer high sensitivity for active viral detection, and biosensors provide quantification, their high manufacturing costs and logistical complexities remain significant barriers in endemic settings. Consequently, the mu3ABC LFA serves as proof that a cost-effective and instrumentation-free platform is still the most practical solution for FMD screening and surveillance.

## 5. Conclusions

The developed mu3ABC strip test demonstrates a reliable diagnostic performance for detecting FMDV NSP-specific antibodies in field conditions. With a diagnostic sensitivity of 98.05% and specificity of 93.16% relative to a commercial ELISA, and substantial agreement (Kappa = 0.690) with the RRL reference standard, the test provides results comparable to established laboratory-based assays. The simplicity, rapid turnaround time, and lack of requirement for specialized equipment of the mu3ABC strip test make it a highly accessible and effective screening platform. By facilitating the differentiation of infected from vaccinated animals (DIVA), this test enables the rapid identification of infected individuals in the field, thereby significantly supporting FMD surveillance and control efforts.

## 6. Patents

A Thai petty patent application based on the results of this study has been submitted (Application No. 2603000415; Filing Date: [30 January 2026]).

## Figures and Tables

**Table 1:** Comparative diagnostic performance of the mu3ABC strip test against a commercial NSP ELISA across three livestock species ^a^.

	ID Screen^®^ FMD NSP ELISA					
	Pigs		Goats		Cattle	
	+	−	+	−	+	−
**mu3ABC strip test** **Positive** **Negative**	38 0	0 99	3 0	9 97	60 2	9 49
**Sensitivity**	100%		100%		96.77%	
**Specificity**	100%		91.51%		84.48%	

^a^ Diagnostic sensitivity and specificity are determined using 366 serum samples, with results compared against the ID Screen^®^ FMD NSP ELISA as the reference standard. Numbers in the cells indicate the count of samples per category (NSP: non-structural protein; +: positive; −: negative).

**Table 2:** Comparison of the mu3ABC strip test performance against a commercial ELISA using the RRL reference standard.

	Reference Standard (RRL)	
	Positive	Negative
**mu3ABC strip test** **Positive** **Negative**	78 22	9 91
**ID Screen^®^ FMD NSP ELISA** **Positive** **Negative**	69 31	0 100

## Data Availability

The original contributions presented in this study are included in the article/Supplementary Materials. Further inquiries can be directed to the corresponding author.

## Supplementary Materials

The following supporting information can be downloaded at https://www.mdpi.com/article/10.3390/vetsci13080797/s1. Figure S1: Complete western blotting illustrations of the purified mu3ABC with FMDV-positive bovine serum. Lanes 1: purified mu3ABC from *E. coli* by ion exchange chromatography and affinity chromatography; Lane 2: purified mu3ABC from *E. coli* by affinity chromatography; Lane 3: purified mu3ABC from insect cells. Blot images, prior to the densitometry readings, were converted to grayscale with ImageJ (ImageJ v. 1.54t, National Institutes of Health) as follows: Image -> Type −> 8 bit. Figure S2: Optimization of key parameters for mu3ABC strip test: (a) the dilution of serum sample at 1:10 dilution, 1:5 dilution, and no serum were tested with 1 µg *E*. *coli* mu3ABC and 1× Protein G conjugated gold nanoparticles; (b) the concentration of mu3ABC at 0.5 µg, 1 µg, and 2 µg were tested with serum sample at 1:10 dilution and 3× Protein G conjugated gold nanoparticles; (c) the amount of protein G-conjugated gold nanoparticles at 2× and 3x were tested with serum sample at 1:10 dilution and 1 µg *E*. *coli* mu3ABC. Upper dot: mouse IgG, lower dot: mu3ABC protein. The signal intensities of the dot blots were quantified using ImageJ software (ImageJ v.154t, National Institutes of Health, USA) by converting digital images to 8-bit grayscale, inverting them, and measuring the Mean Gray Value within a fixed circular Region of Interest (ROI) across three independent replicates (n = 3). The final intensity for each dot was obtained by subtracting the average background intensity measured from three distinct protein-free membrane areas. Table S1: Densitometry readings of the analyzed bands. Table S2: Optimization of experimental parameters for the dot blot assay (n = 3).

## Abbreviations

The following abbreviations are used in this manuscript:

°C	Degree Celsius
µL	Microliter
µg	Microgram
AcMNPV	Autographa Californica Multiple Nucleopolyhedrovirus
AuNPs	Gold Nanoparticles
BSA	Bovine Serum Albumin
CI	Confidence Interval
CRISPR	Clustered Regularly Interspaced Short Palindromic Repeat
DIVA	Differentiation of Infected from Vaccinated Animals
DSn	Diagnostic Sensitivity
DSp	Diagnostic Specificity
EDTA	Ethylenediaminetetraacetic Acid
ELISA	Enzyme-Linked Immunosorbent Assay
FMD	Foot-and-Mouth Disease
FMDV	Foot-and-Mouth Disease Virus
h	Hour
HRP	Horse Radish Peroxidase
IPTG	Isopropyl β-D-1-thiogalactopyranoside
kDa	Kilodalton
LFA	Lateral Flow Immunochromatographic Assay
M	Molar
mA	Milliampere
mg	Milligram
mL	Milliliter
mM	Millimolar
mm	Millimeter
nm	Nanometer
NSP	Non-Structural Protein
OD	Optical Density
POC	Point-of-Care
RRL	Regional Reference Laboratory for Foot and Mouth Disease in South East Asia
RT-LAMP	Reverse-Transcription Loop-Mediated Isothermal Amplification
RT-qPCR	Reverse-Transcription Quantitative Polymerase Chain Reaction
S/N%	Sample-to-Negative Control Percentage
SDS-PAGE	Sodium Dodecyl Sulfate–Polyacrylamide Gel Electrophoresis
TBST	Tris-Buffered Saline with Tween 20
V	Volt
