# Supplementary material for: Development of a mu3ABC-Based Lateral Flow Immunochromatographic Strip for Rapid DIVA-Compatible Detection of Antibodies Specific to Foot-and-Mouth Disease Virus
Source: Vet Sci. 2026 Aug 11;13(8):797. doi: 10.3390/vetsci13080797 (PMC13517950; doi:10.3390/vetsci13080797)
Supplement: Supplementary file 1 [file vetsci-13-00797-s001.zip › vetsci-4463658-supplementary.pdf]

Supplementary data

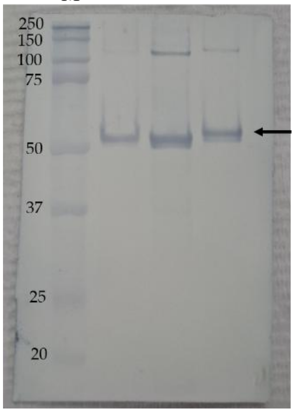

Figure S1 Complete western blotting illustrations of the purified mu3ABC with FMDV-positive bovine serum. Lanes 1: purified mu3ABC from *E. coli* by ion exchange chromatography and affinity chromatography; Lane 2: purified mu3ABC from *E. coli* by affinity chromatography; Lane 3: purified mu3ABC from insect cells. Blot images, prior to the densitometry readings, were converted to grayscale with ImageJ (ImageJ v. 1.54t, National Institutes of Health) as follows: Image -> Type -> 8 bit.

Table S1. Densitometry readings of the analyzed bands.

Lane	Sample Name	Densitometry Reading (Arbitrary Units)	Normalized Intensity Ratio (Relative to Lane 1)
**1**	purified mu3ABC from *E. coli* by ion exchange chromatography and affinity chromatography	221,459.12	1.00
**2**	purified mu3ABC from *E. coli* by affinity chromatography	210,891.24	0.95
**3**	purified mu3ABC from insect cells	191,405.34	0.86

Note: The densitometry analysis was performed using ImageJ software. The normalized intensity ratio was calculated by dividing the reading of each lane by the reading of Lane 1. The primary objective of this gel remains the qualitative verification of the identical molecular weight across all samples.

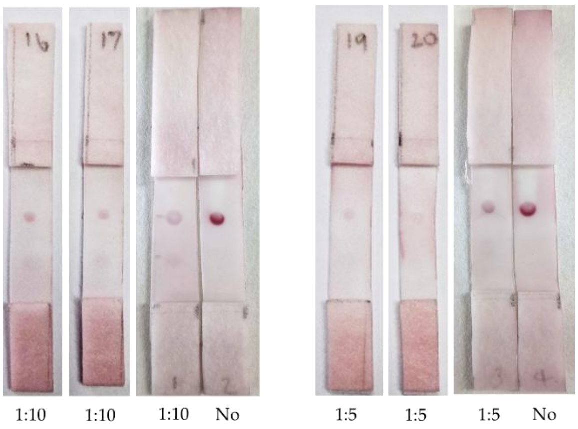

(a)

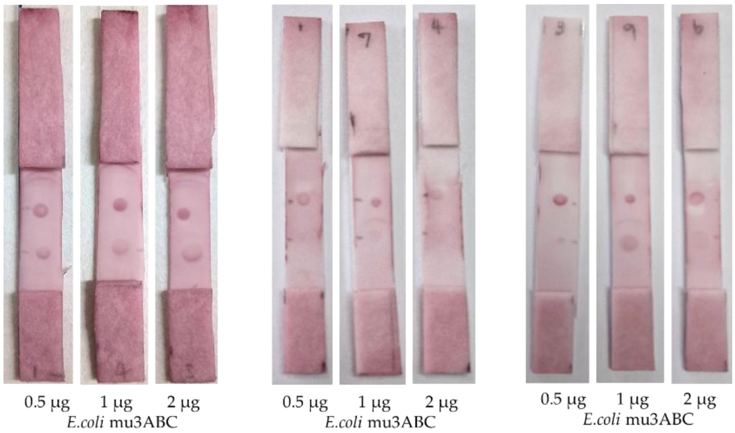

(b)

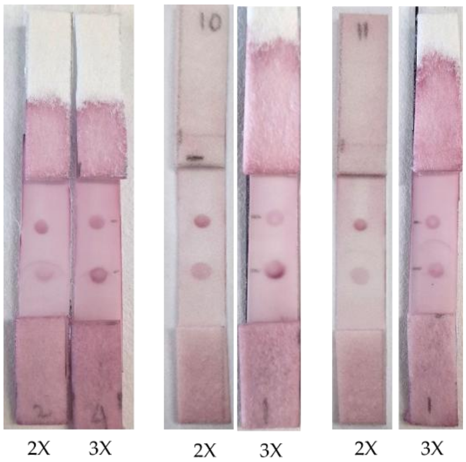

(c)

Figure S2 Optimization of key parameters for mu3ABC strip test: (a) the dilution of serum sample at 1:10 dilution, 1:5 dilution, and no serum were tested with 1 µg *E. coli* mu3ABC and 1x Protein G conjugated gold nanoparticles; (b) the concentration of mu3ABC at 0.5 µg, 1 µg, and 2 µg were tested with serum sample at 1:10 dilution and 3x Protein G conjugated gold nanoparticles; (c) the amount of protein G-conjugated gold nanoparticles at 2x and 3x were tested with serum sample at 1:10 dilution and 1 µg *E. coli* mu3ABC. Upper dot: mouse IgG, lower dot: mu3ABC protein. The signal intensities of the dot blots were quantified using ImageJ software (ImageJ v.154t, National Institutes of Health, USA) by converting digital images to 8-bit gray-scale, inverting them, and measuring the Mean Gray Value within a fixed circular Region of Interest (ROI) across three independent replicates (n = 3). The

final intensity for each dot was obtained by subtracting the average background intensity measured from three distinct protein-free membrane areas.

Table S2 Optimization of experimental parameters for the dot blot assay (n = 3)

Parameter	Condition	Mean Intensity	Standard Deviation (SD)	Statistical Test	*p*-value
**Dilution of serum sample**	1:10 Dilution	15.64	6.57	One-tailed *t*-test	0.033
	1:5 Dilution	1.82	0.72		
**Concentration of mu3ABC**	0.5 mg/mL	13.61	3.74	One-way ANOVA	0.917
	1.0 mg/mL	18.32	14.127		
	2.0 mg/mL	14.89	5.6		
**Amount of protein G-conjugated gold nanoparticles**	2x Test line	14.926	5.28	One-tailed *t*-test	0.038
	3x Test line	26.24	9.57		
	2x Control line	18.07	1.54	One-tailed *t*-test	0.317
	3x Control line	10.99	7.08		
